# Transcranial magnetic stimulation therapy for central post-stroke pain: systematic review and meta-analysis

**DOI:** 10.3389/fnins.2024.1345128

**Published:** 2024-02-14

**Authors:** Francisco Gurdiel-Álvarez, Víctor Navarro-López, Sergio Varela-Rodríguez, Raúl Juárez-Vela, Ana Cobos-Rincón, Juan Luis Sánchez-González

**Affiliations:** ^1^International Doctoral School, Faculty of Health Sciences, Rey Juan Carlos University, Madrid, Spain; ^2^Department of Physical Therapy, Occupational Therapy, Rehabilitation and Physical Medicine, Rey Juan Carlos University, Madrid, Spain; ^3^Cognitive Neuroscience, Pain, and Rehabilitation Research Group (NECODOR), Faculty of Health Sciences, Rey Juan Carlos University, Madrid, Spain; ^4^Department of Physical Therapy, Occupational Therapy, Rehabilitation and Physical Medicine, Rey Juan Carlos University, Madrid, Spain; ^5^Department of Nursing and Physiotherapy, Faculty of Nursing and Physiotherapy, Instituto de Investigación Biomédica de Salamanca (IBSAL), Salamanca, Spain; ^6^Nursing Department, Faculty of Health Sciences, University of La Rioja, Research Group GRUPAC, Logroño, Spain

**Keywords:** meta-analysis, stroke, transcranial magnetic stimulation, pain management, pain

## Abstract

**Introduction:**

Although rare, central post-stroke pain remains one of the most refractory forms of neuropathic pain. It has been reported that repetitive transcranial magnetic stimulation (rTMS) may be effective in these cases of pain.

**Aim:**

The aim of this study was to investigate the efficacy of rTMS in patients with central post-stroke pain (CPSP).

**Methods:**

We included randomized controlled trials or Controlled Trials published until October 3rd, 2022, which studied the effect of rTMS compared to placebo in CPSP. We included studies of adult patients (>18 years) with a clinical diagnosis of stroke, in which the intervention consisted of the application of rTMS to treat CSP.

**Results:**

Nine studies were included in the qualitative analysis; 6 studies (4 RCT and 2 non-RCT), with 180 participants, were included in the quantitative analysis. A significant reduction in CPSP was found in favor of rTMS compared with sham, with a large effect size (SMD: −1.45; 95% CI: −1.87; −1.03; *p* < 0.001; I2: 58%).

**Conclusion:**

The findings of the present systematic review with meta-analysis suggest that there is low quality evidence for the effectiveness of rTMS in reducing CPSP.

**Systematic review registration:**

Identifier (CRD42022365655).

## Introduction

According to the International Association for the Study of Pain (IASP), neuropathic pain is any pain caused by a lesion or disease of the somatosensory system (Jensen et al., 2011; Treede et al., 2015). Central post-stroke pain (CPSP) is defined by the IASP as ‘pain initiated or caused by a primary lesion or dysfunction of the central nervous system (IASP, 2011) and occurs in the absence of other nociceptive, peripheral and psychogenic pain (Şahin-Onat et al., 2016).

A recent meta-analysis involving a total of 69 studies by Liampas et al. (2020) estimated that approximately 1 in 10 of all stroke patients will experience neuropathic pain. Other studies (Bowsher et al., 1993; Stitik et al., 2005) indicate that in the USA, the prevalence of CPSP reported in one study ranged from 2 to 8% in 250,000 people who suffered a cerebrovascular accident in the course of 1 year. Other authors widen the range even further, establishing a prevalence between 1 and 35% (Oh and Seo, 2015). This broad estimate is possibly due to variabilities in the definition of this pain category, the inclusion criteria, and the length of patients’ evaluation post-stroke (Kumar et al., 2009).

Once stroke patients overcome the acute phase of the event, they need early neurorehabilitation treatment to alleviate the consequences of the injury, such as spasticity or CSPS. Currently, there is no globally accepted and approved pharmacological therapy to accelerate the recovery of these patients (Figueroa et al., 2015). Therefore, new therapies, such as transcranial magnetic stimulation (TMS), have emerged. TMS consist of a high voltage and high intensity discharge system attached to a transducing coil. This system generates short lasting (<1 ms) magnetic fields of 1–2.5 Tesla, which penetrates the skull and induces secondary electric currents in the cerebral cortex that depolarizes neurons (Groppa et al., 2012). This phenomenon could be used as evaluation tool to assess corticospinal pathway integrity, applying a single pulse at cortical level and registering electric activity at the motor end-plate (Spampinato et al., 2023). Also, it could be used to evaluate intra-cortical excitability changes applying paired pulses with different time intervals (Wagle-Shukla et al., 2009).

For CPSP treatment one of the most used TMS techniques is repetitive transcranial magnetic stimulation (rTMS). rTMS is a noninvasive brain stimulation technique that generates brief, rapidly changing magnetic fields capable of inducing electric currents in the brain (Young et al., 2014). It is safe, well tolerated, and has a very favorable side effect profile, provided that safety recommendations are followed (Burke et al., 2019). Depending on stimulation parameters, rTMS can have an excitatory or inhibitory effect on the underlying neural networks (Young et al., 2014). At frequencies ≥5 Hz (high-frequency rTMS), rTMS has been shown to produce an increment in cortical excitability in healthy humans (Fitzgerald et al., 2006) and stroke patients (Belardinelli et al., 2021). This improvement in cortical excitability is the result of a modulation of the GABAergic and glutamatergic systems (Esser et al., 2006; Belardinelli et al., 2021) producing a long-term potentiation phenomenon in the stimulated neural networks (Esser et al., 2006). Moreover, ≤1 Hz frequencies (low-frequency rTMS) produce the opposite effect via long-term depression (Chen et al., 1997). These neuroplastic changes could induce reorganization of neural networks in the motor cortex, supplementary motor area, premotor area, cerebellum, thalamus and corpus callosum (Tosun et al., 2017; Guo et al., 2021; Wanni Arachchige et al., 2023). As well as reversal of functional connectivity changes (Grefkes et al., 2010; Guo et al., 2021; Juan et al., 2022) that occur after the stroke (Li et al., 2017; Vecchio et al., 2019).

According to the scientific literature, rTMS has numerous applications as analgesic tool in different neuropathic pain conditions. A long lasting analgesic effect has been reported when applying 5 sessions of high-frequency rTMS in CPSP or trigeminal neuralgia patients, compared to sham stimulation (Khedr et al., 2005). This reduction in pain intensity is also observed in other neuropathic pain conditions after receiving a rTMS treatment (Ahmed et al., 2011; Attal et al., 2021). In the only one systematic review conducted on the effect of non-invasive brain stimulation on CPSP, Ramger et al. (2019) concluded that noninvasive brain stimulation can have a therapeutic effect on the pain level of people with CPSP, as evidenced by significant decreases in clinical and experimental pain scores. Although no more systematic reviews or meta-analysis have been conducted on the analgesic effect of rTMS on CPSP, we can observe the same effect across other chronic neuropathic pain conditions (Gatzinsky et al., 2021).

To date no quantitative synthesis of the effect of rTMS on CPSP has been performed. Therefore, the aim of this study was to perform a meta-analysis of the randomized clinical trials (RCTs) or non-randomized clinical trials (CTs) that investigated the efficacy of rTMS in patients with CPSP.

## Methods

Guidelines from the Preferred Reporting Items for Systematic Review and Meta-analysis (PRISMA) statement were consulted to develop this systematic review (Moher et al., 2010). The computerized databases Medline (Pubmed), SCOPUS, Cochrane Library, Embase, and Web of Science were used to search for relevant studies. Keywords referring to the intervention were used, combined with Boolean operators (complete search strategy is shown in Supplementary Appendix S1).

Searches were performed between September 3rd 2022, and October 3rd 2022, (from the date of inception of each database) using a combination of controlled vocabulary (i.e., medical subject headings) and free-text terms. Search strategies were modified to meet the specific requirements of each database. Searches of the reference lists of included studies and previously published systematic reviews were also conducted.

This meta-analysis was registered in the International Prospective Register of Systematic Reviews (PROSPERO registration no: CRD42022365655).

### Criteria for considering studies and study selection

We used the Population, Intervention, Comparison, Outcomes, Time, and Study design (PICOTS) as a framework to formulate eligibility criteria (Lira and Rocha, 2019).

### Population

Individuals diagnosed with CPSP secondary to an ischemic or hemorrhagic stroke in the central nervous system.

### Intervention

Treatment must consist of the application of at least one session of rTMS in the motor cortex.

### Comparison

Comparison groups could be another type of intervention or non-intervention.

### Outcomes

The measurement used to assess the outcomes and effects of the exercise was pain intensity. Measurements were to be recorded by objective methods, using validated and reliable scales or questionnaires (e.g., pain intensity by visual analog scale or numerical rating scale). Variables were to be assessed before and after the intervention.

### Time

No temporal restrictions were applied to the duration of the intervention or outcome measures.

### Studies

Only RCTs or CTs were included.

### Data extraction

At first, two blinded investigators (JLS-G and FG-A) examined the studies obtained from the databases by screening by title and abstract according to the established inclusion criteria. In the case of discrepancies, a third investigator (SV-R) intervened. After this first screening, the selected articles were read in full to see if they definitely met the criteria and could be included in the analysis. The authors of the included studies were contacted by e-mail, with the aim of accessing possible unclear data. If no response was received, the data were excluded from the analysis.

### Risk of bias and assessment of methodological quality of the studies

Two reviewers independently assessed the risk of bias in the studies (FGA and JLSG).

The risk of bias in non-randomized studies of interventions (NRSI) was assessed through the Risk of Bias In Non-randomized Studies of interventions (ROBINS-I) (Sterne et al., 2016). This tool focuses on assessing the risk of bias (RoB) in the results of NRSIs. The types of NRSIs that can be assessed with this tool are quantitative studies estimating the efficacy (harm or benefit) of an intervention, which did not use randomization to assign units (individuals or groups of individuals) to comparison groups. ROBINS-I takes into account 6 domains: Randomization process (D1), Bias arising from period and carryover effects (DS), Deviations from the intended interventions (D3), missing outcome data (D4), Selection of the reported result (D5).

On the other hand, a revised tool to assess the risk of bias in randomized clinical trials (RoB2) (Higgins et al., 2011) was used to assess the risk of bias in randomized trials. The tool is structured into five domains through which bias could be introduced into the outcome. These were identified based on empirical evidence and theoretical considerations. Because the domains cover all types of bias that may affect the results of randomized trials, each domain is mandatory, and no additional domains should be added. The five domains for individually randomized trials (including crossover trials) are: bias arising from the randomization process (D1); bias due to deviations from intended interventions (D2); bias due to missing outcome data (D3); bias in the measurement of the outcome (D4); bias in the selection of the reported result (D5).

In addition, methodological quality was evaluated using the PEDro list (de Morton, 2009), which assesses the internal and external validity of a study and consists of 11 criteria: (1) specified study eligibility criteria; (2) random allocation of subjects; (3) concealed allocation; (4) measure of similarity between groups at baseline; (5) subject blinding; (6) therapist blinding; (7) assessor blinding; (8) fewer than 15% dropouts; (9) intention-to-treat analysis; (10) between group statistical comparisons; and (11) point measures and variability data. The methodological criteria were scored as follows: yes (one point), no (zero points), or unknown (zero points). The PEDro score of each selected study provided an indicator of the methodological quality (9–10 = excellent; 6–8 = good; 4–5 = fair; 3–0 = poor) (Higgins et al., 2011).

### Overall quality of the evidence

The overall quality of evidence was based on the classification of the results into levels of evidence according to the Grading Of Recommendations Assessments, Development, and Evaluation (GRADE), which is based on 5 domains: (1) Study design; (2) Imprecision; (3) Indirectness; (4) Inconsistency; (5) Publication bias.

Evidence was categorized into the following 4 levels accordingly: (a) High quality: further research is very unlikely to change our confidence in the estimate of effect, all 5 domains are also met; (b) Moderate quality: further research is likely to have an important impact on our confidence and might change the estimate of effect, one of the 5 domains is not met; (c) Low quality: further research is very likely to have an important impact on our confidence and is likely to change the estimate of effect, two of the 5 domains are not met; and (d) Very low quality: any estimate of effect is very uncertain, 3 of the 5 domains are not met (Balshem et al., 2011; Andrews et al., 2013).

### Data synthesis and analysis

Meta-analysis was performed using ReviewManager statistical software (version 5.4; Cochrane, London, UK). Effects were investigated by calculating standardized mean differences (SMDs) for change scores from baseline to intervention. For this, the sample size, mean difference, and standard deviations (SDs) were extracted. When the study only reported median and first and third quartile values, they were converted to means and SDs (Luo et al., 2018).

When the authors presented only standard errors, these were converted to SDs. If the study did not present the results, the authors were contacted to request them. If results were not available in this way, means and SDs were estimated from graphs (Image J program; National Institute of Health in Bethesda, Maryland, United States). If none of this was possible, the study was excluded from the quantitative analysis and the information was presented narratively.

If the study did not report the preintervention postintervention mean difference in each group, the mean difference was obtained using the pre-postintervention values. In the absence of SD of the difference, we imputed from other data reported in the study: (1) using other measures reported in the study (e.g., confidence intervals and *p* values, following the principles described in Chapter 6.5.2.2 of the Cochrane Handbook) (Higgins et al., 2023); or, if that was not possible, (2) using the correlation coefficient of the most similar study included (following the principles described in Chapter 6.5.2.8 of the Cochrane Handbook) (Higgins et al., 2023); or if that was not possible, (3) using a conservative correlation coefficient of 0.5 (Deeks et al., 2022). This methodology has been performed in other meta-analyses (Gurdiel-Álvarez et al., 2023).

Meta-analysis was performed using the inverse variance method and a random effects model with 95% confidence intervals, as it provides more conservative results in case of heterogeneity between studies, which is expected. *p* values < 0.05 were considered statistically significant. An effect size (SMD) of 0.8 or greater was considered large, an effect size between 0.5 and 0.8 was considered moderate, and an effect size between 0.2 and 0.5 was considered small.

A sensitivity analysis was performed to evaluate the results. For this purpose, the meta-analysis was performed only with studies with low RoB, and then with the correlation coefficient of 0.5, instead of being estimated from the other studies. Sensitivity analysis was performed when the analysis could be performed in at least 5 studies. Study heterogeneity was assessed by the degree of between study inconsistency (I2). The Cochrane Group has established the following interpretation of the I2 statistic: 0–40% may not be relevant/important heterogeneity, 30–60% suggests moderate heterogeneity, 50–90% represents substantial heterogeneity, and 75–100% represents considerable heterogeneity (Balk et al., 2012). Skewness was assessed using funnel plots according to application method (cathodic, anodic), and stimulation site. These analyses were performed only if the subgroups had at least three studies.

### Inter-rater reliability

Inter-rater reliability for screening, risk of bias assessment, and quality of the evidence rating were assessed using percentage agreement and Cohen’s kappa coefficient (Cohen, 1968; McHugh, 2012). There was strong agreement between reviewers for the screening records and full texts (98.51% agreement rate and *k* = 0.91), the risk of bias assessment (92.86% agreement rate and *k* = 0.83), and the quality and strength of the evidence assessment (97.73% rate and *k* = 0.95).

## Results

**Figure 1:**
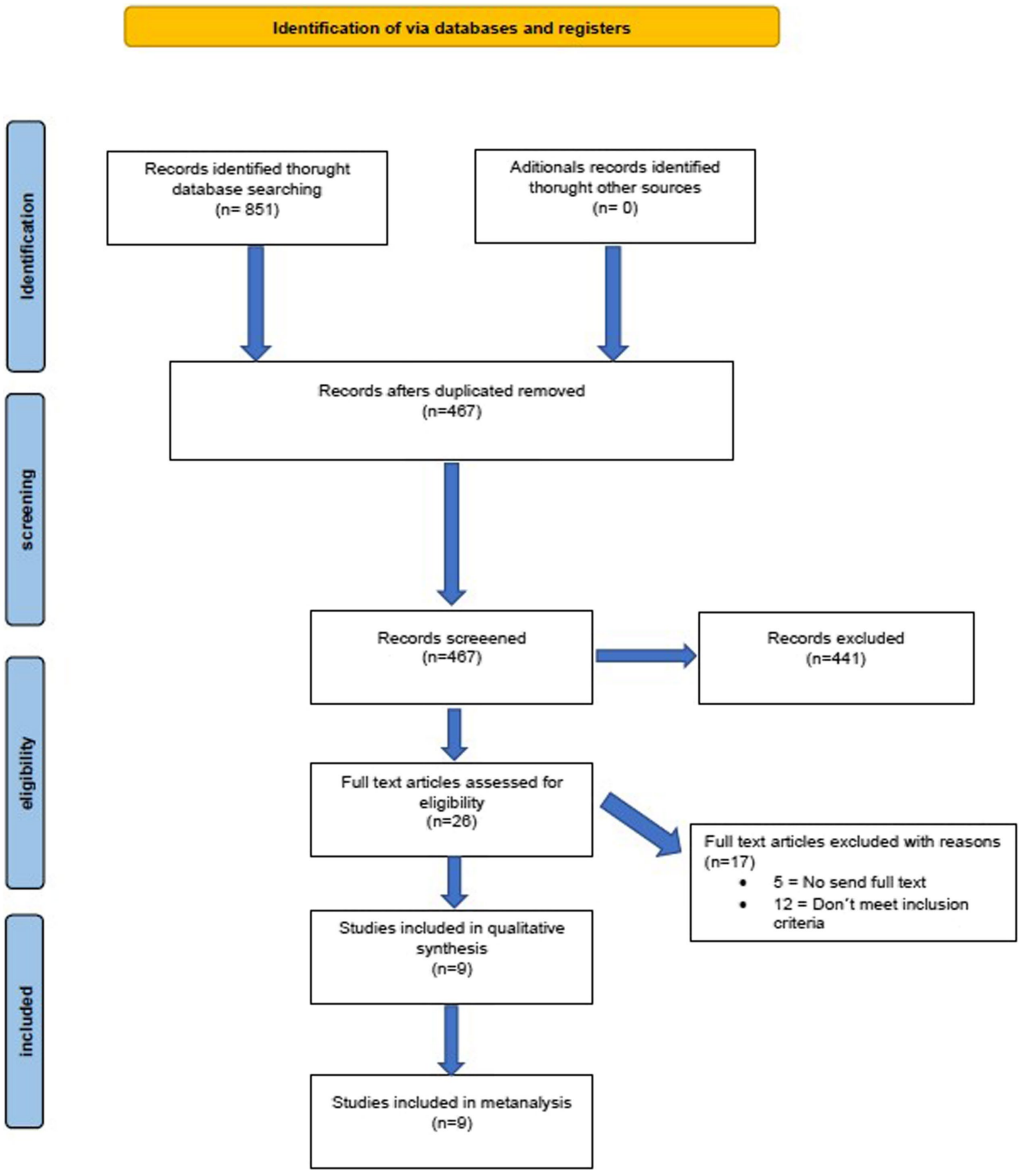
Flowchart.

The search found 851 records, of which 384 were duplicates and 467 were screened by title and abstract. Twenty five studies were potentially relevant and full reports were obtained and screened. Seventeen studies were justifiably excluded. Nine studies met the eligibility criteria and were included for review (Figure 1).

### Characteristics of included studies

Nine studies (180 participants; 78 women) were included for review (Table 1). Six were RCTs and three were CTs. The mean age of participants was 56.73 ± 9.78 years. Mean pain duration was 39.08 ± 23.42 months. Mean pain intensity was 66.57 ± 12.20 in a 0 to 100 scale.

**Table 1:** Studies characteristics.

Study	Design	Group (sample size)	Gender, male (female)	Age, years	Pain duration (months)	Localization of injury (*n*)	Etiology of injury	Stimulation site	Adverse effects	Stimulation protocol	Pain outcome
Hosomi et al. (2013)	RCT (cross-over)	G1	16 (13)	61.5 ± 10.9	56.4 ± 63.1	Thalamus (15) Lenticular nucleus (6) Subcortex (1) Other (7)	NR	M1 contralateral corresponding to painful site	Deterioration of squeezing (3%), deterioration of numbness (1%) and hypoglycemia (1%)	A stimulation session was carried out daily for 10 consecutive days. A real rTMS session consisted of 10 trains at 90% intensity of resting motor threshold (one train, 50 pulses at 5 Hz; intertrain interval, 50 s). A total of 500 pulses were applied in a session	VAS SF-MPQ
		G2	14 (11)	60.1 ± 10.5	59.5 ± 47.0	Thalamus (14) Lenticular nucleus (12) Subcortex (2) Brain stem (2) Other (5)				A stimulation session was carried out daily for 10 consecutive days. Ten trains of electrical stimuli at 2 times the intensity of the sensory threshold (one train, 50 stimuli at 5 Hz; intertrain interval, 50 s) were delivered with a conventional electrical stimulator through the electrodes fixed on the head	
Khedr et al. (2005)	CT	G1	14 (Sex distribution NR)	52.3 ± 10.3	18 ± 17	Thalamic infarction (12) Thalamic hemorrhage (6) Parietal infarction (2) Other (4)		M1 contralateral of abductor digiti minimi	NR	Real-rTMS involved applying a train of rTMS once per minute for 10 min. Each train consisted of 200 pulses at 20 Hz and 80% RMT (total duration of 10 s) applied through a figure of eight coil over the identified motor cortical area corresponding to the hand of the painful side. The treatment was repeated every day for five consecutive days	VAS
		G2	10 (Sex distribution NR)							Sham-rTMS was applied using the same parameters but with the coil elevated and angled away from the head to reproduce some of the subjective sensation of rTMS and yet avoid induction of current in the brain	
Kobayashi et al. (2015)	RCT (cross-over)	G1	4 (2)	63 ± 9.9	9 ± 6.83		Ischemic (5) Hemorrhagic (11)	M1 contralateral to most painful arm/leg	Transient slight scalp discomfort	Real focal 5 Hz rTMS was delivered to the scalp over the primary motor cortex of the affected hemisphere. The intensity of rTMS was set at 90% of the active motor threshold for the targeted hemisphere. Real rTMS involved a train of 50 pulses at 5 Hz (total duration 10 s). The train was repeated ten times, and a total of 500 pulses were delivered over a 10-min session, with a 50-s inter-train interval	VAS
		G2								Sham rTMS was performed with the coil held at an angle of 90° to the scalp using the same stimulation parameters (noise, time, frequency) as those for real rTMS.	
Lefaucheur et al. (2001a)	RCT (cross-over)	G1	6 (8)	57.2	NR	NR	NR	M1 contralateral to painful site	None	For “real” TMS a series of 20 trains of 5 s in duration (55-s intertrain interval) at a stimulation rate of 10 Hz and 80% of rest motor threshold intensity	VAS
		G2								The same protocol was used for sham stimulation, but using a “sham” 8-shaped coil	
Lefaucheur et al. (2001b)	RCT (cross-over)	G1	11 (7)	54. 7	NR	thalamic (6) Brain Stem (6) brachial plexus (6)	NR	M1 contralateral to painful site	None	(1) a series of 20 10 Hz trains of 5 s duration (55 -s intertrain interval) at 80% of rest motor threshold intensity using a real TMS coil; (2) the same protocol using a sham 8-shaped coil (Magstim Placebo Coil System 1730-23-00, The Magstim Co., Whitland, UK); (3) a 20 min stimulation at 0.5 Hz and at 80% of rest motor threshold intensity using a real TMS coil	VAS
Matsumura et al. (2013)	CT (cross-over)	G1	12 (8)	63.6 ± 8.1	2.95 ± 1.36	Thalamic (11) Putamen (5) Brainstem (4)	Ischemic (7) Hemorrhagic (13)	M1 contralateral to most painful site	NR	For rTMS, the subjects sat relaxed on a stimulation chair while a total of 500 stimuli at 5 Hz were delivered to the part of the motor cortex that corresponded to the site of most severe pain on the lesion side. The stimulation intensity was 100% resting motor threshold of the unaffected primary motor cortex of the hand area, with 50 pulses per train at 25-s intertrain intervals	VAS
		G2								Sham rTMS was performed under the same conditions, but the stimulation coils were elevated at an angle of 45° from the skull	
André-Obadia et al. (2006)	RCT (cross-over)	G1	10 (4)	53 ± 11	82.8 ± 48	Brainstem (10) Other (4)		M1 contralateral to painful site	None	Cortical inhibitory stimulation at M1: 1 Hz repetitive stimulation at 90% of motor threshold during 26 min, i.e., a total of 1,600 stimulations	VAS
		G2								Cortical excitatory stimulation at M1: 20 consecutive trains of 80 stimulations at 20 Hz (90% motor threshold), separated by inter-trains intervals of 84 s, i.e., a total of 1,600 stimulations	
		G3								Sham stimulation at M1: same protocol as 1 Hz stimulation using the coil oriented perpendicular to, and separated from, the skull, thus preventing actual cortical stimulation	
Ojala et al. (2022)	RCT (cross-over)	G1	8 (9)	55.8 ± 7.1	NR	NR	Ischemic (10) Hemorrhagic (7)	M1 contralateral representation of the abductor pollicis brevis of the painful site	Headache (*n* = 1) Tiredness (*n* = 2) Paresthesia (*n* = 2) Transient increase in pain (*n* = 2) Collapse (*n* = 1)	The nrTMS was applied at 10 Hz during a 50-min period with an intensity of 90% of the MT. Altogether, 5,050 pulses per session were given in trains of 101 pulses (10-s stimulation with a 50-s intertrain interval). The electric fields induced by the nrTMS ranged from 31 to 127 V/m in the underlying M1 cortex	NRS
		G2						S2 in the parietal operculum lateral upper lip of the Sylvian fissure	Headache (*n* = 3) Tiredness (*n* = 3) Paresthesia (*n* = 3) Transient increase in pain (*n* = 3) Increase spasticity (*n* = 2) Dizziness (*n* = 1)	The nrTMS was applied at 10 Hz during a 50-min period with an intensity of 90% of the MT. Altogether, 5,050 pulses per session were given in trains of 101 pulses (10-s stimulation with a 50-s intertrain interval) The corresponding values in the chosen lateral cortical site for S2 ranged from 39 to 109 V/m	
		G3						Same as M1 group	Headache (*n* = 4) Tiredness (*n* = 2) Paresthesia (*n* = 3) Transient increase in pain = 2	Sham nrTMS was delivered over the M1 cortex by attaching a 75-mm non-conductive plastic block on the coil to increase the coil-to-scalp distance and to minimize the electric field induced in the cortex	
Zhao et al. (2021)	RCT	G1	19 (Sex distribution NR)	50.16 ± 11.34	0.2 ± 0.10	Right cerebellum (1) Right thalamus (4) Right basal ganglia (7) Right external capsule (2) Right lateral periventricular (1) Right frontal lobe (2) Left cerebellum (1) Left thalamus (4) Left frontal lobe (2) Left basal ganglia (10) Left external capsule (1) Left lateral periventricular (3)	Ischemic (17) Hemorrhagic (21)	M1 contralateral to painful site	Numbness in the scalp or twitching of facial muscles during procedure (*n* = 3)	In the active rTMS stimulation group, rTMS was applied over the motor cortical area (M1) corresponding to the painful zone at a frequency of 10 Hz, as 15 pulse trains (1.5 s), with intertrain intervals of 3 s (total of 1,500 pulses) and at an intensity of 80% of the RMT (AH), or 100% (UH) when the RMT could not be detected in the AH	NRS SF-MPQ2
		G2	19 (Sex distribution NR)	48.95 ± 11.51	0.21 ± 0.41					The sham stimulation was delivered using a coil identical to the one in the active group (same shape and color), but with no magnetic stimulation output (only emitting the same sound) Patients in the active rTMS and sham groups received stimulation once a day, 6 days per week, for a total of 3 weeks	

### Quality assessment

Methodological quality scores ranged from 3 to 10 out of a maximum of 10 points. Three studies (33%) were of high methodological quality (greater than or equal to 6 points). Table 2 show the details of the PEDro scale.

**Table 2:** Methodological score of randomized clinical trials using the Physiotherapy Evidence Database (PEDro) scale.

Study	1	2	3	4	5	6	7	8	9	10	11	Total
Hosomi et al. (2013)	Y	Y	Y	Y	Y	N	Y	Y	Y	Y	Y	10
Khedr et al. (2005)	N	N	N	Y	Y	N	Y	N	N	Y	N	4
Kobayashi et al. (2015)	N	N	N	N	Y	N	N	Y	N	Y	N	3
Lefaucheur et al. (2001a)	N	Y	N	Y	N	N	N	Y	N	Y	N	4
Lefaucheur et al. (2001b)	N	Y	N	Y	N	N	N	Y	Y	Y	N	5
Matsumura et al. (2013)	N	N	N	Y	N	N	N	Y	N	Y	N	3
André-Obadia et al. (2006)	N	Y	N	N	Y	N	Y	N	N	Y	N	4
Ojala et al. (2022)	Y	Y	Y	Y	Y	N	Y	N	N	Y	N	7
Zhao et al. (2021)	Y	Y	Y	Y	Y	N	Y	Y	N	Y	Y	9

Y: yes; N: no. 1: eligibility criteria specify; 2: random allocation of participants; 3: concealed allocation; 4: similarity between groups at baseline; 5: participant blinding; 6: therapist blinding; 7: assessor blinding; 8: dropout rate less than 15%; 9: intention-to-treat analysis; 10: between-group statistical comparisons; 11: point measures and variability data.

### Risk of bias

**Figure 2:**
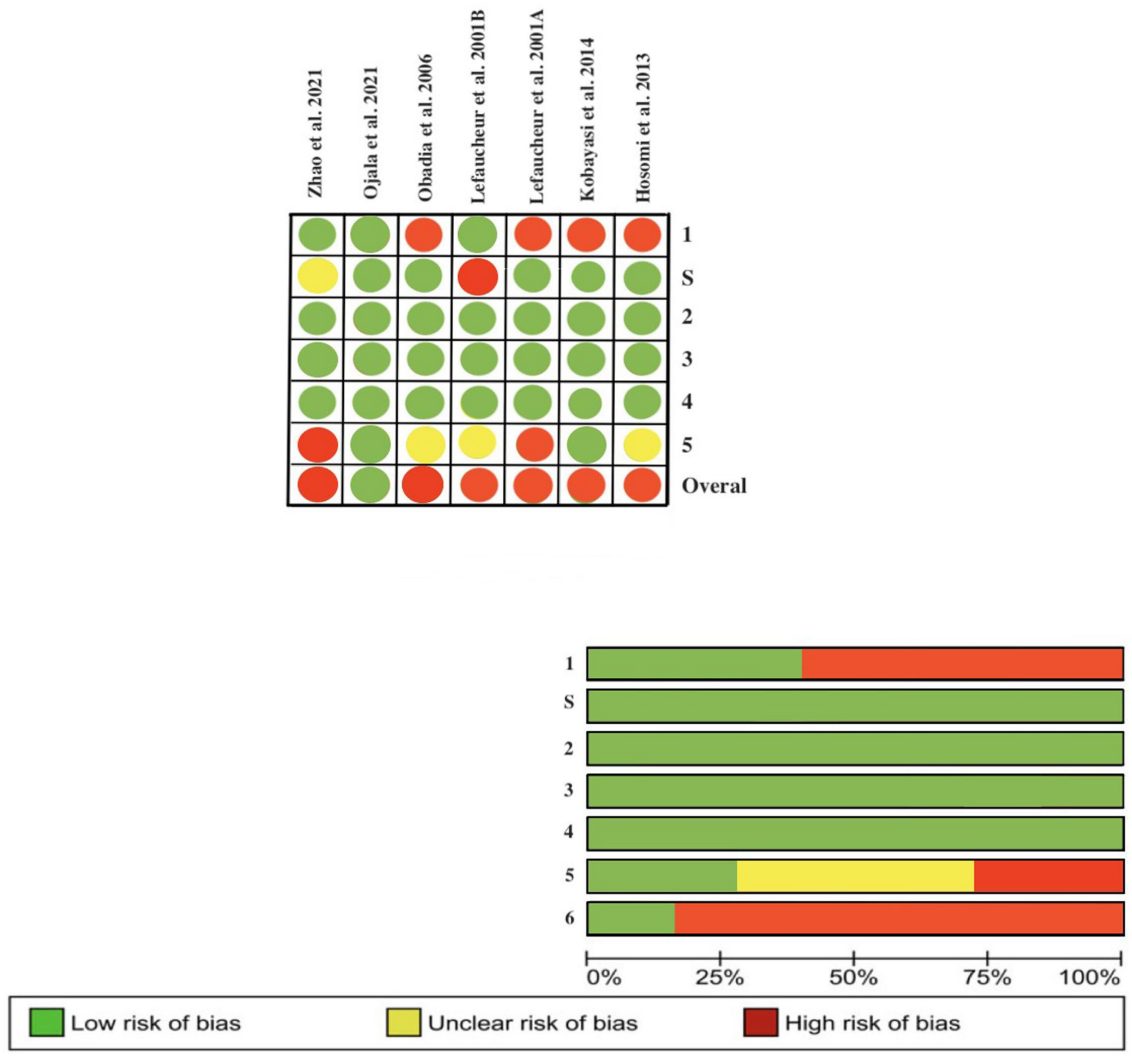
Assessment of the risk of bias according to the revised Cochrane risk-of-bias tool for randomized trials (ROB-2).

**Figure 3:**
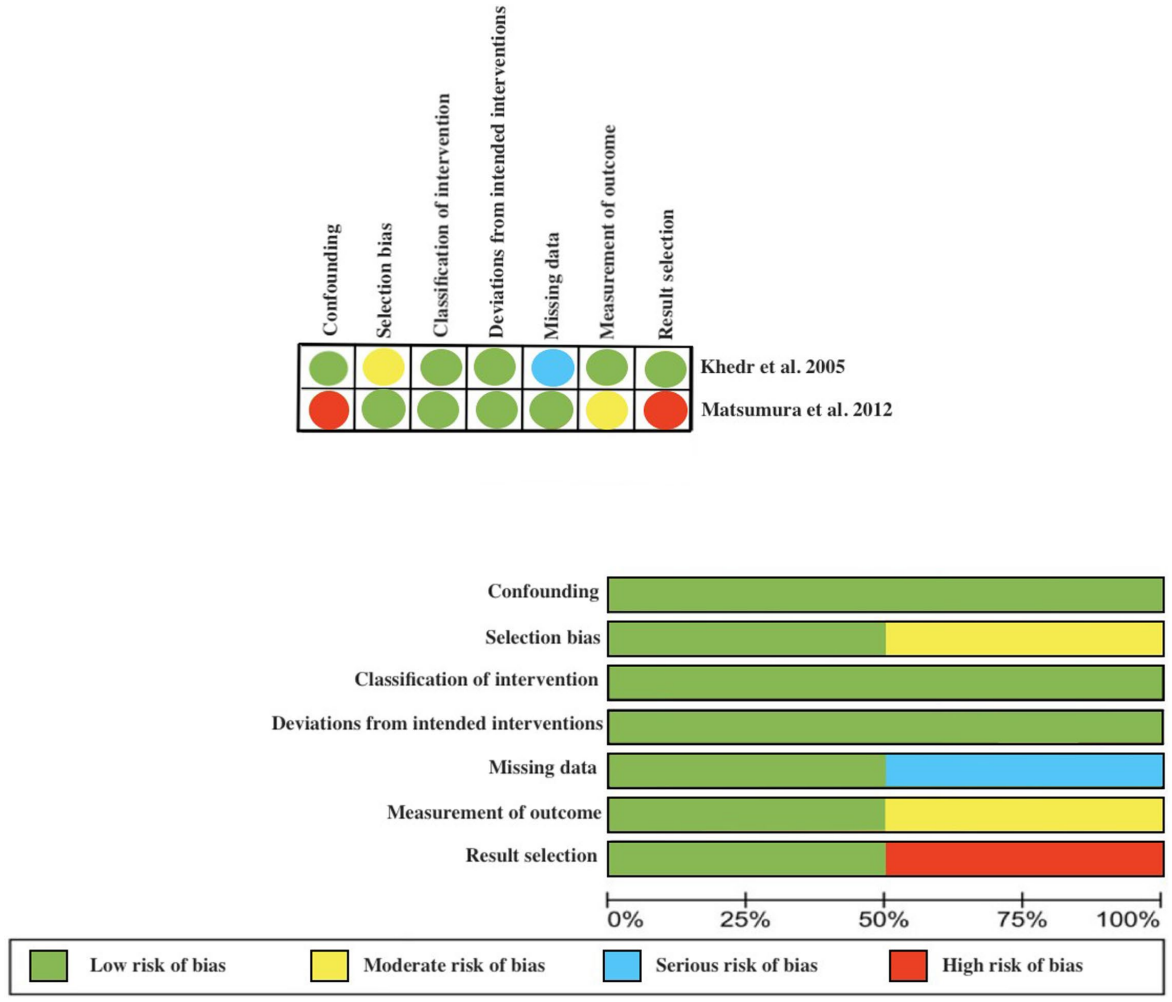
Assessment of the risk of bias according to the Robins scale.

We assessed the quality of the included studies using the RoB 2 tool for the RCTs and the ROBINS-E tool for the non-randomized clinical trials. We only judged 1 study to be at low risk of bias. The majority of the RCTs presented limitations in the randomization process or the report of the outcomes. While in the CTs, there was a risk of selection bias. Assessment of the risk of bias in the included studies is shown in Figures 2, 3.

### Effects of rTMS on neuropathic pain

**Figure 4:**
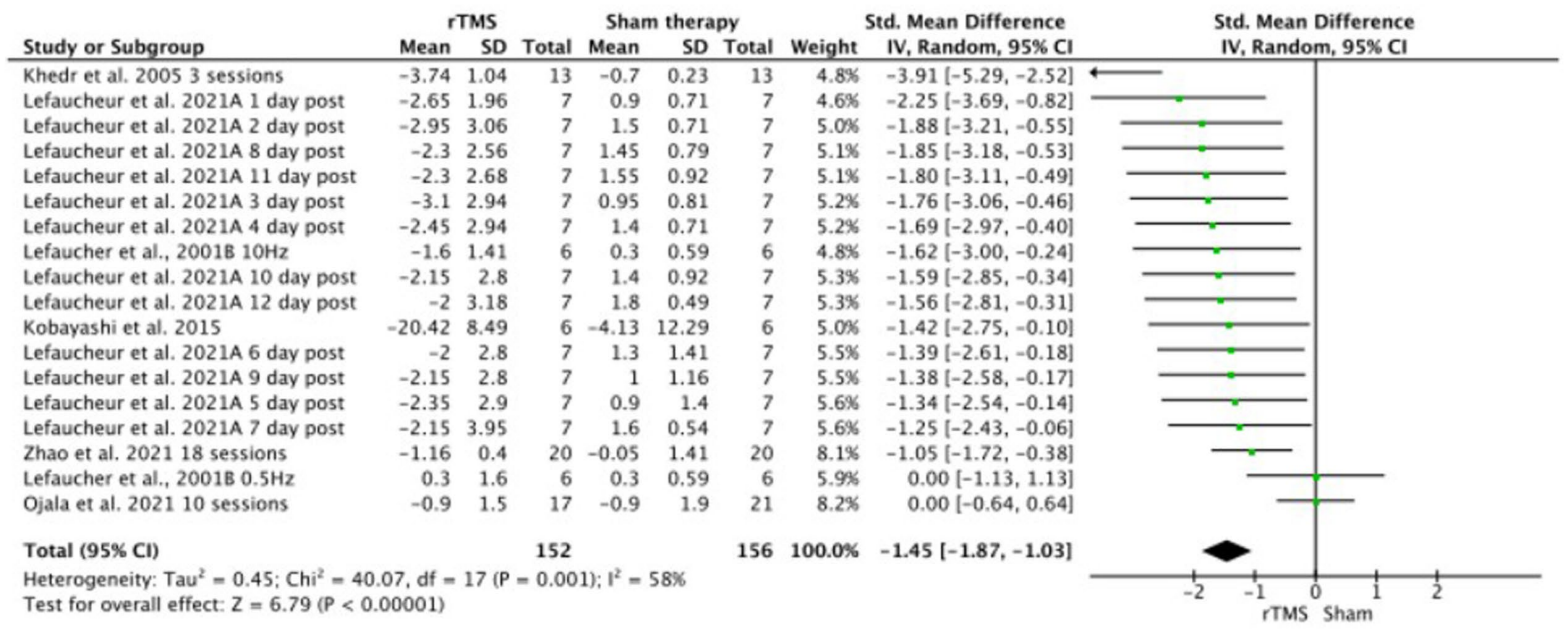
rTMS versus sham forest plot. Forest plot of the results of a random-effects meta-analysis shown as standardized mean differences (SMD) with 95% confidence interval (CI) for the effects of rTMS compared with sham in post-stroke central pain. The shaded square represents the point estimate for each individual study and the study weight in the meta-analysis. The diamond represents the overall mean difference of the studies.

**Figure 5:**
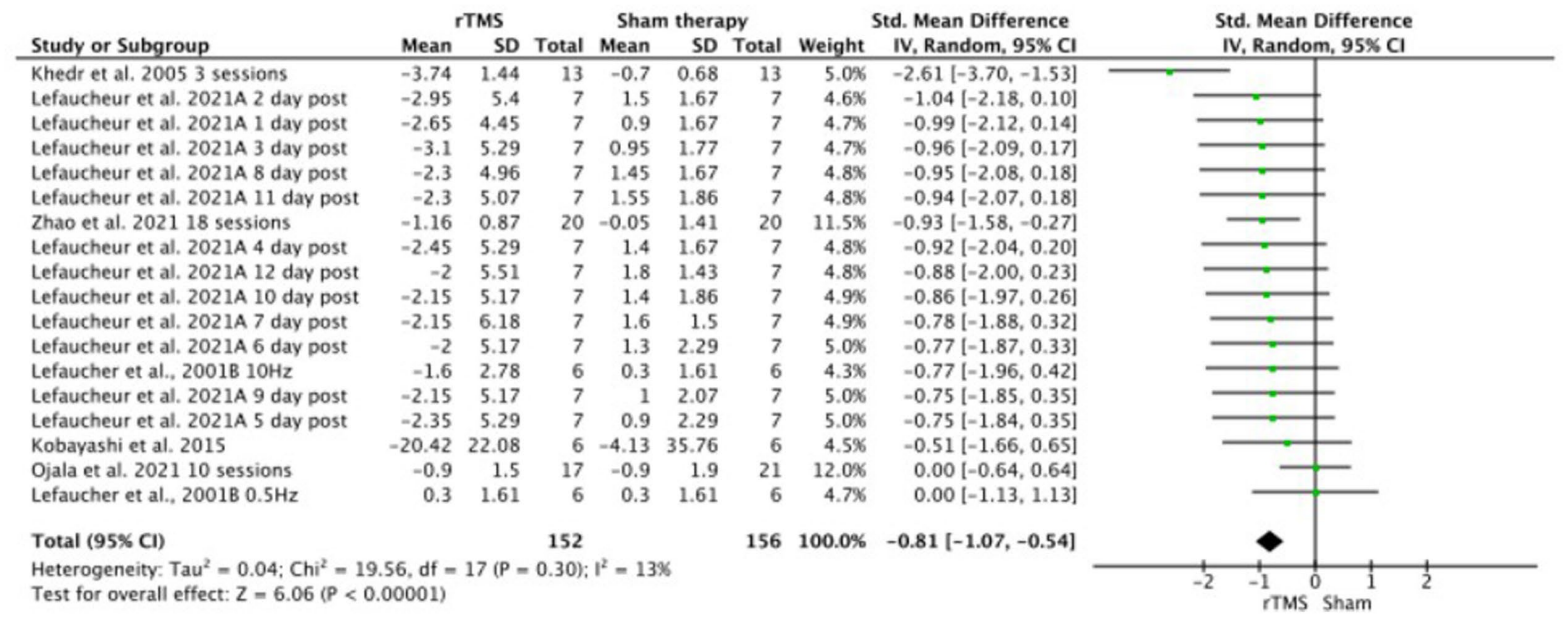
Sensitivity analysis of rTMS versus sham forest plot. Forest plot of the results of a random-effects meta-analysis shown as standardized mean differences (SMD) with 95% confidence interval (CI) for the effects of rTMS compared with sham in post-stroke central pain. The shaded square represents the point estimate for each individual study and the study weight in the meta-analysis. The diamond rep-resents the overall mean difference of the studies.

**Figure 6:**
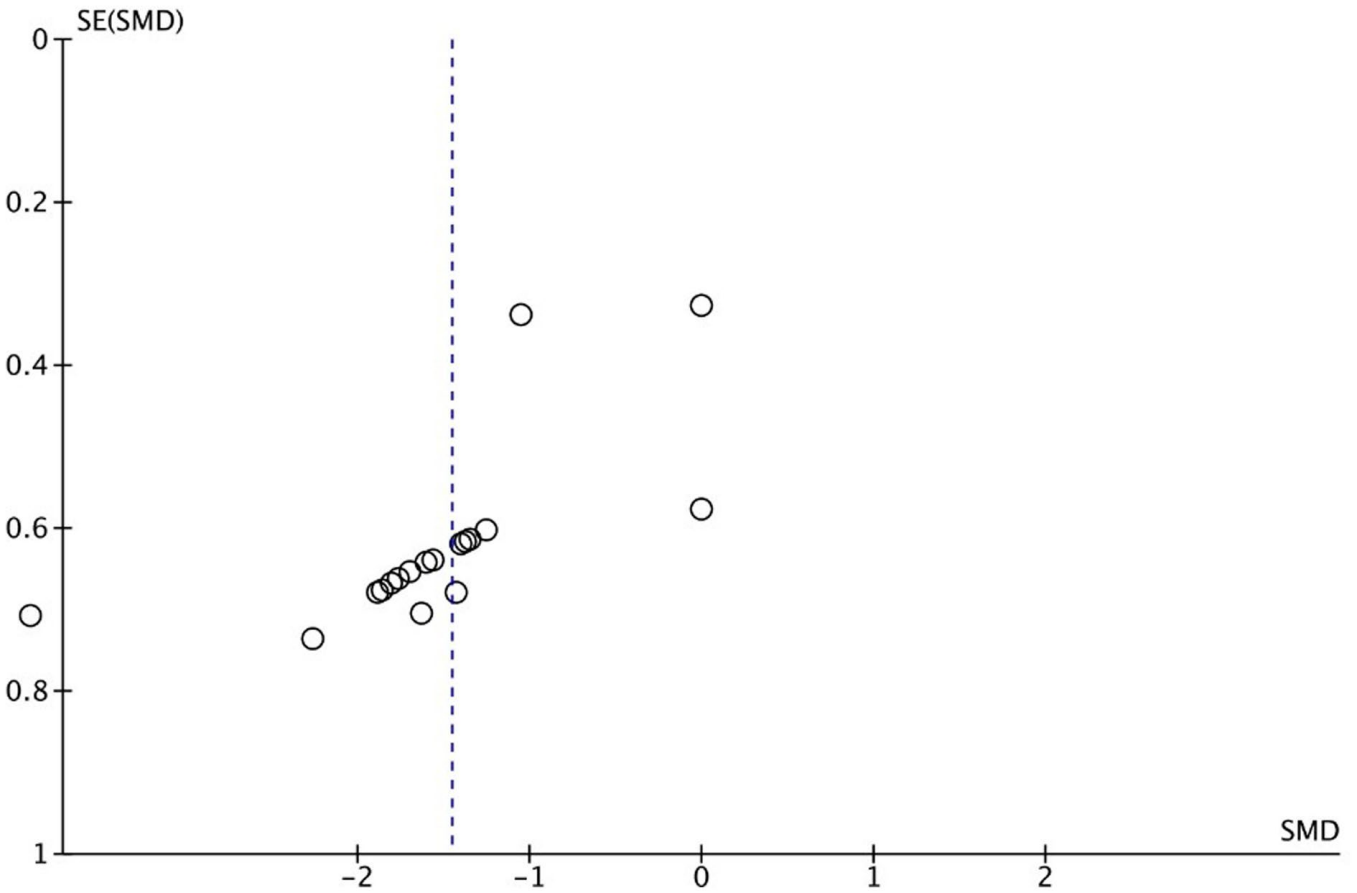
Central post-stroke pain funnel plot.

Meta-analysis showed that significantly (*p* < 0.001), rTMS-based intervention produces a reduction in pain compared to sham based interventions with a large effect size (SMD: −1.45; 95% CI: −1.87; − 1.03; *Z*: 6.79; *p* < 0.001), and a moderate- substantial heterogeneity (*I*^2^: 58%; *p* = 0.001) (Figure 4). Sensitivity analysis by RoB could not be performed since only one study showed a low risk of bias. In the sensitivity analysis, a conservative correlation coefficient of 0.5 was applied, instead of being estimated from the other studies, the effect size was reduced from large to medium, but the significance remained in favor of the rTMS (SMD: −1.45; 95% CI: −1.87; −1.03; Z: 6.79; *p* < 0.001 to SMD: −1.81; 95% CI: −1.07; −0.54; *Z*: 6.06; *p* < 0.001). Heterogeneity was reduced from *I*^2^: 58%; *p* = 0.001 to *I*^2^ = 13%; *p* = 0.3 (Figure 5). The funnel plot presents asymmetry, indicating the risk of publication bias (Figure 6).

### Adverse effects of intervention

Two non-randomized clinical trials did not mention adverse effects when reporting their results (Khedr et al., 2005; Matsumura et al., 2013). In one RCT, two patients reported transient, slight scalp discomfort after real rTMS (Kobayashi et al., 2015). Another RCT reported mild and transient adverse effects, such as headaches, tiredness, paresthesia, transient increase of pain, collapse, increased spasticity, or dizziness (Ojala et al., 2022). Lastly, other RCT reported short periods of numbness in the scalp or twitching of the fascial muscle during the stimulation in three participants (Zhao et al., 2021). The rest of the studies reported no adverse effects experienced during the duration of the intervention or follow-up (Lefaucheur et al., 2001a,b; André-Obadia et al., 2006; Hosomi et al., 2013).

### Quality of evidence

Table 3 collects the details of the GRADE assessment. Three levels of evidence were downgraded due to the serious inconsistency of the results, publication bias, and overall risk of bias, which suggests a very small level of evidence regarding the effects of rTMS in patients with CPSP.

**Table 3:** GRADE evidence for rTMS to treat central post-stroke pain.

Number of studies	Risk of bias*	Inconsistency^†^	Indirectness^‡^	Imprecision^§^	Publication Bias^¶^	MD or SMD (95% CI)	Quality of evidence
Five trials (*n* = 197)	Serious (mainly by blinding the therapist)	Serious (*I*^2^ = 67%)	No serious	No serious	Serious	MD = −1.65 (−2.46, −0.84) SMD = −1.21 (−1.95, −0.47)	Very small

GRADE, Grading of Recommendations Assessment, Development and Evaluation; MD, mean difference; SMD, standardized mean difference.*“No” = most information is from results at low risk of bias; “Serious” = crucial limitation for one criterion or some limitations for multiple criteria sufficient to lower confidence in the estimate of effect; “Very serious” = crucial limitation for one or more criteria sufficient to substantially lower confidence in the estimate of effect.^†^“Serious” = *I*^2^ > 40%; “Very serious” = *I*^2^ > 80%.^‡^No indirectness of evidence was found in any study.^§^Based on sample size. “Serious” = *n* < 250 subjects; “Very serious” = *n* < 250 and the estimated effect is little or absent.^¶^Based on funnel plots. No publication bias was found. Funnel plots are not shown because the number of trials was less than 10.

## Discussion

This systematic review included nine studies, of which six were RCTs, the largest cohort of clinical studies on the effects of rTMS on CPSP. This meta-analysis of data from six trials provides very small level of evidence of a large effect size on pain reduction when active rTMS is delivered on affected M1 in CPSP compared to a sham intervention. However, after carrying a sensitivity analysis, the effect size of the intervention is determined to be moderate with a low heterogeneity.

To date, this is the first meta-analysis evaluating the analgesic effect of rTMS on CPSP. Other studies have reviewed the antalgic effect of non-invasive physical modalities on CPSP, including rTMS. In other systematic review (Chen et al., 2016), a reduction of 10.8–32.6% in pain intensity was found in four non-randomized controlled trials (Hirayama et al., 2006; Goto et al., 2008; Ohn et al., 2012; Hosomi et al., 2013) and 1 case study (Lefaucheur et al., 2004) but no effect was seen in 2 RCTs (Lefaucheur et al., 2001b; de Oliveira et al., 2014). As a result, a Level B of evidence was given to rTMS as an analgesic tool for the treatment of CPSP (Chen et al., 2016). Another systematic review evaluated the effect of non-invasive brain stimulation on CPSP (Ramger et al., 2019). It found that of the 5 studies about rTMS (Ohn et al., 2012; Matsumura et al., 2013; de Oliveira et al., 2014; Hasan et al., 2014), 2 RCTs (Matsumura et al., 2013; Kobayashi et al., 2015) and 2 non-randomized clinical trials (Ohn et al., 2012; Hasan et al., 2014) reported a decrease in pain intensity after the treatment with high-frequency rTMS on the affected hemisphere (Ramger et al., 2019).

After the stroke, there is a remapping of the motor cortex that has been observe by functional magnetic resonance imaging (fMRI) or TMS (Cicinelli et al., 1997, 2003; Traversa et al., 1997). In animal stroke models, we can observe a significant reduction of the affected area in the motor cortex (Nudo, 2007). In addition, neuronal connections on the side contralateral to the lesion appear to be altered resulting in a lateralization of the neural activity (Bütefisch et al., 2008). In consequence, a decrease in short interval intracortical inhibition in both hemispheres, and an increase in intracortical facilitation in the non lesioned hemisphere can be observed (Liepert et al., 2000; Swayne et al., 2008). This results in an imbalance in the interhemispheric inhibition that could result in an obstacle for recovery (Vallone et al., 2016).

With regard to CPSP treatment, motor cortex stimulation has been researched since 1991 (Tsubokawa et al., 1991) as an invasive procedure to treat drug-resistant central pain. In motor cortex stimulation, higher frequencies are used, and the electrode is implanted in the affected hemisphere. High-frequency rTMS of the affected hemisphere tends to be the most common type of stimulation seen in CPSP trials, whereas low-frequency rTMS of the affected hemisphere is less common and tends to not have an effect (Lefaucheur et al., 2001a). Several mechanisms for high-frequency rTMS modulation of CPSP have been proposed (Pan et al., 2023; Radiansyah and Hadi, 2023). Electrical stimulation of the motor cortex increases blood flow to the lateral thalamus, the anterior cingulate cortex, the anterior insula, and the brainstem of CPSP patients (García-Larrea et al., 1999). Similar patterns of activity had been reported on fMRI after rTMS of M1 (Bestmann et al., 2004), implying common mechanisms of action. This analgesic effect of motor cortex stimulation in CPSP patients seems to be determined by the availability of opioid receptors in the anterior cingulate cortex, the insula, the thalamus, and the periaqueductal gray matter (Maarrawi et al., 2013). Meaning that rTMS of affected M1 in CPSP patients could modulate these structures of the medial system of pain (Xie et al., 2009), which has been shown to mediate the affective processing of the pain experience (Vogt and Sikes, 2000). Regarding this, animal CPSP models exhibit a reduction in the number of fibers in the thalamocortical pathway between the ventral posterolateral nucleus of the thalamus and the somatosensory cortices (Kadono et al., 2021) and increased functional connectivity between the medial thalamus and the amygdala (Mitchell and Chakraborty, 2013). The analgesic effect of rTMS in these models is associated with a reduction in the strength of the functional connectivity between medial thalamus and amygdala, normalizing during the rTMS treatment (Kadono et al., 2021).

Another proposed mechanism is the increase in excitability of the affected M1, that seems to be reduced in CPSP patients as a result of an asymmetric interhemispheric inhibition (Pan et al., 2023; Radiansyah and Hadi, 2023). The lesion of one M1 reduces its inhibitory activity in the contralateral M1. This results in an increase on the excitability of the contralateral M1 and a higher inhibitory output from the contralateral M1 to the injured M1 (Boddington and Reynolds, 2017). The application of high frequency rTMS to the injured M1 produce an increment on the excitability of the affected cortex, and an inhibition of the augmented excitability of the contralateral M1 (Bai et al., 2022). Finally, the activation of the descending inhibitory system is another mechanism that could explain the analgesic effect of non-invasive brain stimulation (DosSantos et al., 2018). However, in CPSP patients, heterotopic noxious conditioning stimulation, which activates the descending inhibitory system, has failed to reduce ongoing pain and dynamic mechanical allodynia (Tuveson et al., 2009).

Pharmacological treatment of the CPSP tend to use some drugs that could interact with the mechanism of action of TMS, and therefore alter its effects (Ziemann et al., 2015). Amitriptyline is used as first line treatment for CPSP (Ziemann et al., 2015), but its interaction with TMS is not known. It acts inhibiting voltage gated ion channels (Yan et al., 2010; Wu et al., 2012) and could act as agonist of TrkA and TrkB receptors, which mediate neural plasticity (Jang et al., 2009). Also, it seems to decrease GABAergic transmission (Bang et al., 2021). These mechanisms could potentially result in the increase of the facilitatory effect of the high frequency rTMS, and in the decrease of the inhibitory effect of the low frequency rTMS. Anticonvulsants like gabapentin or pregabalin, are other type of drugs that have been implemented in the management of CPSP (Hesami et al., 2015). Gabapentin and pregabalin have been shown to block voltage-gated ion channels, increase the synthesis and brain concentrations of GABA (Löscher et al., 1991) and reduce the synaptic release of glutamate (Taylor et al., 2007). These effects could produce an increase in the motor threshold measured by TMS (Menzler et al., 2014), a more sustained intracortical inhibition (Rizzo et al., 2001; Sommer et al., 2012) and a diminished intracortical facilitation (Rizzo et al., 2001). Accordingly, to this, stroke patients receiving anticonvulsant treatment could benefit less from high frequency rTMS treatment.

Considering the results of the present systematic review with meta-analysis, rTMS could be considered useful tool in the clinical context for management CPSP. Not only it has several possible mechanisms of action on the pathophysiological processes underlying CPSP as previously presented (Bestmann et al., 2004; Bai et al., 2022; Pan et al., 2023; Radiansyah and Hadi, 2023), but it is also a less invasive treatment that motor cortex stimulation (Tsubokawa et al., 1991). Also, high frequency rTMS protocols last only about 10 min and its adverse effects tend to be rare and mild in nature. Due to its suitability for the clinical practice, future studies should consider evaluating rTMS effectiveness compared to other treatments recommended for the management of patients with CPSP (e.g., adrenergic antidepressants or anticonvulsants) or its interaction with them.

### Strengths and limitations

Several limitations should be kept in mind when interpreting the results of the meta-analysis. Two of the studies included in the meta-analysis were not RCTs, so there exists some risk of selection bias. Regarding the duration of pain, some studies did not report it, while others ranged between acute (<3 months) to chronic presentation (>3 months). Mixing patients with acute and chronic CPSP in the study sample could account to an increased variability in the results, due to differences in the underlying pathophysiological processes. So future studies should consider these differences when stablishing their inclusion criteria. Also, the dosage of the rTMS varied between studies, with the frequency of stimulation ranging between 5 and 20 Hz, the intensity of stimulation ranging between 80 and 100% resting motor threshold, and the total number of sessions ranging between 1 and 18 sessions. Analyzing together studies with different rTMS protocols could in fact account to differences in the measured effects, accounting to increased heterogeneity in the results. Due to scarcity in studies applying same rTMS protocols in CPSP, future studies should take into account replicating the methodology of stimulation of previous studies to reduce this problem. Lastly, there seems to be a common risk of bias between the included studies regarding the randomization process or the clarity in the report of the outcomes. Researchers must consider reporting clearly the randomization processes to reduce possible biases and facilitate replicability, as well as expressing measures of centralization and dispersion to improve transparency and better understanding of the results.

The mains strengths of this study, is that this is the first systematic review with meta-analysis that has investigated the efficacy of rTMS on patients with CPSP. An analysis methodology has been applied in which pre-post mean differences were compared, which provides robustness to the results. The analysis has been developed based on the most recommended guidelines so that the study is replicable. The sensitivity analysis allowed to reduce the heterogeneity of the analyzed data sample, increasing the robustness of the results. Future studies should aim to improve the randomization and blinding processes to reduce the risk of bias, and define better the characteristics of the included subjects to provide homogeneous samples.

## Conclusion

The findings of the current systematic review with meta-analysis suggest that there is low quality evidence for the effectiveness of rTMS in reducing CPSP intensity with a large effect size. Future studies should consider improving methodology by blinding the therapist and taking into account patients’ characteristics and rTMS parameters to reduce heterogeneity.

## Data availability statement

The original contributions presented in the study are included in the article/Supplementary material, further inquiries can be directed to the corresponding author.

## Author contributions

FG-Á: Conceptualization, Methodology, Supervision, Writing – original draft. VN-L: Data curation, Formal analysis, Investigation, Software, Writing – original draft. SV-R: Investigation, Validation, Visualization, Writing – review & editing. RJ-V: Data curation, Project administration, Writing – review & editing. AC-R: Data curation, Formal analysis, Resources, Validation, Writing – review & editing. JS-G: Conceptualization, Investigation, Methodology, Supervision, Writing – original draft.
