# Supplementary material for: Transcranial magnetic stimulation therapy for central post-stroke pain: systematic review and meta-analysis
Source: Front Neurosci. 2024 Feb 14;18:1345128. doi: 10.3389/fnins.2024.1345128 (PMC10899389; doi:10.3389/fnins.2024.1345128)
Supplement: Supplementary file 1 [file Table_1.DOCX]

| **Appendix 1:** Database formulas during literature search |
| --- |
| **PubMed Search Formula: 119**  (“Stroke” OR “Cerebral Stroke”) AND (“transcranial magnetic stimulation”) AND (“Pain” OR “Pain referred”) |
| **Cochrane Library Search Formula: 62**  (“Stroke” OR “Cerebral Stroke”) in Title Abstract Keyword AND (“transcranial magnetic stimulation”) in Title Abstract Keyword AND (“Pain” OR “Pain referred”) in Title Abstract Keyword |
| **SCOPUS Search Formula: 219**  TITLE-ABS-KEY (“Stroke” OR “Cerebral Stroke”) AND (“transcranial magnetic stimulation”) AND (“Pain” OR “Pain referred”) |
| **WOS Search Formula: 224** |

(“Stroke” OR “Cerebral Stroke”) AND (“transcranial magnetic stimulation”) AND (“Pain” OR “Pain referred”)

**Embase Search Formula: 227**

(“Stroke” OR “Cerebral Stroke”) AND (“transcranial magnetic stimulation”) AND (“Pain” OR “Pain referred”)
